# Targeted protein depletion in *Saccharomyces cerevisiae *by activation of a bidirectional degron

**DOI:** 10.1186/1752-0509-4-176

**Published:** 2010-12-29

**Authors:** Marc Jungbluth, Christian Renicke, Christof Taxis

**Affiliations:** 1 Department of Genetics, Philipps-Universität Marburg, Karl-von-Frisch-Str. 8, 35043 Marburg, Germany

## Abstract

**Background:**

Tools for *in vivo *manipulation of protein abundance or activity are highly beneficial for life science research. Protein stability can be efficiently controlled by conditional degrons, which induce target protein degradation at restrictive conditions.

**Results:**

We used the yeast *Saccharomyces cerevisiae *for development of a conditional, bidirectional degron to control protein stability, which can be fused to the target protein N-terminally, C-terminally or placed internally. Activation of the degron is achieved by cleavage with the tobacco etch virus (TEV) protease, resulting in quick proteolysis of the target protein. We found similar degradation rates of soluble substrates using destabilization by the N- or C-degron. C-terminal tagging of essential yeast proteins with the bidirectional degron resulted in deletion-like phenotypes at non-permissive conditions. Developmental process-specific mutants were created by N- or C-terminal tagging of essential proteins with the bidirectional degron in combination with sporulation-specific production of the TEV protease.

**Conclusions:**

We developed a system to influence protein abundance and activity genetically, which can be used to create conditional mutants, to regulate the fate of single protein domains or to design artificial regulatory circuits. Thus, this method enhances the toolbox to manipulate proteins in systems biology approaches considerably.

## Background

One of the basic experiments in cell biology is to remove a specific protein, conduct a phenotypic analysis and from that deduce the function of the protein. Often, the main problem is to remove the protein of interest quickly and efficiently. Many techniques have been developed to disturb the synthesis, activity or abundance of a selected protein [1-7]. The addition of a destabilizing sequence (degron) is used to reduce the half-life of the target protein by inducing proteasomal proteolysis [8]. A recent development to target proteins for degradation is the auxin-inducible degron, a relative of the PROTACS system. Both methods use chemical compounds to tether a protein to an ubiquitin-protein-ligase, thereby inducing polyubiquitylation and degradation of the target protein [9,10]. Other methods utilize small chemical compounds to change the destabilization activity of protein domains [11,12], or degrade target proteins employing the bacterial protease ClpXP [13].

One very widespread method is the release of an N-degron by proteolysis applying the ubiquitin-fusion technique. The fusion of ubiquitin to the N-terminus of a protein results in cleavage by deubiquitylating enzymes between ubiquitin and the protein. Thereby, almost all amino acids can be exposed at the N-terminus of a protein. The technique led to the formulation of the N-end rule, which states that the *in vivo *half-life of a protein is related to the identity of the N-terminal amino acid [14-16]. In yeast 12 amino acids (D, E, F, H, I, K, L, N, Q, R, W, and Y) destabilize a protein if exposed at the N-terminus. Eight of these destabilizing amino acids are directly recognized by the ubiquitin-protein-ligase Ubr1 (primary destabilizing residues), whereas four have to be modified by deamidation and/or arginylation (tertiary and secondary destabilizing residues) before. Recognition by Ubr1 leads to polyubiquitylation of the protein followed by proteasomal proteolysis [17,18]. Varshavsky and his co-workers developed a temperature-sensitive degron for the creation of conditional mutants [19]. This technique has been used effectively to investigate functions of many essential proteins in *Saccharomyces cerevisiae*, although it is often necessary to use high level expression of *UBR1 *to induce a temperature-sensitive phenotype [20-24].

Recently, the tobacco etch virus (TEV) protease induced protein instability (TIPI) system was developed as another tool to create conditional mutants. A degradation tag (TDegF) with a dormant N-degron is fused to the N-terminus of a target protein. Presence of the TEV protease leads to site-specific proteolysis of the tag and exposure of the N-degron. This initiates destabilization of the target protein. Fast and efficient cleavage of the substrate is achieved utilizing a TEV protease (named pTEV^+ ^protease) with enhanced processivity towards the TDegF tag. Thus, by regulation of *pTEV^+ ^protease *expression the TIPI system allows to control target protein abundance [25].

One of the well studied degrons, which might be useful for protein depletion, is the C-terminal degron of the mouse ornithine decarboxylase (cODC) which is conserved in vertebrates [26]. It induces rapid, proteasomal proteolysis independent of polyubiquitylation [27]. The half-life of full-length mouse ornithine decarboxylase expressed in yeast has been found to be 10 minutes [28]. Two elements within the degron mediate the destabilization activity. The first is a cysteine-alanine motif, which is important for proteasomal association. The second consists of an unstructured region flanking the cysteine-alanine motif, comprising 16 amino acids upstream and 19 downstream [26,29]. The cODC degron destabilizes a protein only if present at the very C-terminus [30]. Moving the cysteine-alanine motif only a few amino acids closer to the C-terminus or farther away impairs the destabilizing activity [26]. The cODC degron has been used to destabilize proteins in the fungus *Saccharomyces cerevisiae *[28], the plant *Nicotiana tabacum cv. Xanthi *[31], mammalian cell culture [32] or to study protein degradation *in vivo *and *in vitro *[33,34], though no conditional version of the degron has been developed. We reasoned that it should be possible to create a conditional C-degron by constructing a tag with the cODC degron kept inactive due to a protective peptide added C-terminally to the cODC degron. Activation of the tag is achieved by site-specific proteolysis resulting in activation of the cryptic C-degron. We chose a dormant N-degron [25] as protective group. Thus, we created a bidirectional degron consisting of two degrons, which inactivate each other until TEV protease cleavage. This bidirectional degron can be fused to the target N-terminally, C-terminally or placed internally to regulate protein abundance *in vivo*.

## Results and Discussion

### Construction of conditional C-degrons

**Figure 1:**
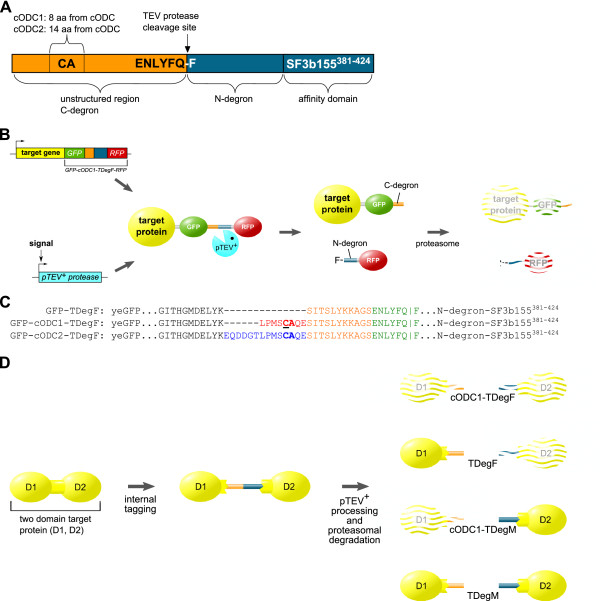
**The bidirectional degron approach enhances the TEV protease induced protein instability (TIPI) system**. **(A) **Schematic illustration of the bidirectional degrons. The degrons consist of an unstructured domain (orange) with cODC sequences (8 or 14 amino acids containing the cysteine-alanine (CA) motif), the TEV protease recognition site (ENLYFQ-F) and an N-degron fused to the affinity domain SF3b155^381-424 ^(blue). The affinity domain binds to the human protein p14, which was fused to the TEV protease. Usage of these affinity domains enhances cleavage of the degradation tags. **(B) **Regulation of target protein abundance by the TEV protease induced protein instability (TIPI) system using the bidirectional degron fused to the C-terminus of the target. Details can be found in the text. **(C) **Sequence of the GFP-TDegF tag with cODC insertions. Amino acids derived from yeast enhanced GFP [64] are shown in black - cODC1 (red) or cODC2 (blue) - spacer sequence (orange) - TEV protease recognition site (green). The cysteine-alanine motif is marked by bold letters; the cysteine in the cODC1 construct, which was mutated to alanine to verify proteasomal degradation is underlined; the TEV protease cleavage site is indicated (|); sequences of the N-degron and SF3b155^381-424 ^can be found in Figure S3 (see Additional file 1) or [25]. **(D) **Control of single domain stability by internal tagging of a two domain target protein. The TIPI system offers modules with stabilizing or destabilizing sequences flanking the TEV protease recognition site. The target protein is tagged between two domains (D1, D2). Depending on the modules used for modification of the target, the domains are destabilized or remain stable after cleavage. Phenylalanine and cODC1 confer destabilization, whereas methionine and unmodified TDeg should not influence protein stability. All possible combinations are shown; features of the different constructs are summarized in Table 1.

The structure of the bidirectional degrons is shown in Figure 1A. We chose the tobacco etch virus (TEV) protease to activate the C-degrons due to our previous experience with the development of the TEV protease induced protein instability (TIPI) system [25]. The degrons are based on the conditional N-degron TDegF, which consists of an unstructured spacer region, the TEV protease cleavage site ENLYFQ-F, an N-degron and an affinity domain derived from the human protein SF3b155 (amino acids 381 to 424). This affinity domain binds strongly to the human protein p14, which is fused to the TEV protease, thereby enhancing processivity of the protease towards the TDegF tag [25]. The bidirectional degrons were created by insertion of sequences containing the cysteine-alanine motif and surrounding amino acids (8 or 14) of the C-terminal mouse ornithine decarboxylase degron (cODC) into the unstructured spacer region in front of the TEV protease recognition site. In both constructs, the cysteine-alanine motif was placed 19 amino acids upstream of the cleavage site. Thus, the newly developed bidirectional degrons consist of a C-degron separated from an N-degron by the TEV protease recognition site (Figure 1A). Due to this arrangement, both degrons are kept inactive until the tag is cleaved by the TEV protease. The usage of the bidirectional degron for conditional destabilization of target proteins is illustrated in Figure 1B: The *GFP-cODC1-TDegF-RFP *tag is fused to the 3'- end of the target gene. Expression of the *pTEV^+ ^protease *gene is controlled by an appropriate promoter (inducible, active during a specific cell-cycle stage or developmental process). Upon expression of the *pTEV^+ ^protease*, the tag is cleaved at the consensus site (ENLYFQ-F) if it is exposed to the cytoplasm or the nucleus. This cleavage leads to activation of the two dormant degrons resulting in rapid proteasomal proteolysis of the target protein and the RFP. The sequences of the N-degron TDegF as well as the bidirectional degrons cODC1-TDegF and cODC2-TDegF are given in Figure 1C.

Due to the modular design of our constructs, the TEV protease cleavage site might be flanked by stabilizing or destabilizing sequences. Depending on the modules used, internal tagging of a two domain protein followed by *in vivo *proteolytic cleavage leads to destabilization of the whole protein, destabilization of one domain or separation of the domains (Figure 1D). Using this strategy, the TIPI system is expanded not only by a C-degron, but gains the possibility to control the fate of protein domains after separation. The features of the newly developed constructs are summarized in Table 1.

**Table 1:** Tags for protein degradation or cleavage

Constructs	N-degron	C-degron	Bidirectional degron	Source
TDegF	**+**	**-**	**-**	[25]

TDegM	**-**	**-**	**-**	[25]

GFP-cODC1-TDegF-RFP	**+**	**+**	**+**	This work

GFP-cODC1-TDegF	**+**	**+**	**+**	This work

cODC1-TDegM	**-**	**+**	**-**	This work

cODC2-TDegF	**+**	**+**	**+**	This work

The sequence of TDegM is similar to TDegF with the exception of the TEV protease cleavage site (ENLYFQ-M replaces ENLYFQ-F). The sequences for cODC1-TDegF and cODC2-TDegF differ in the length of the cODC sequence inserted into TDegF (Figure 1A, C). The ability to act as an N-degron, a C-degron or a bidirectional degron is indicated for all constructs (+).

### Rapid protein depletion induced by the cODC degron

**Figure 2:**
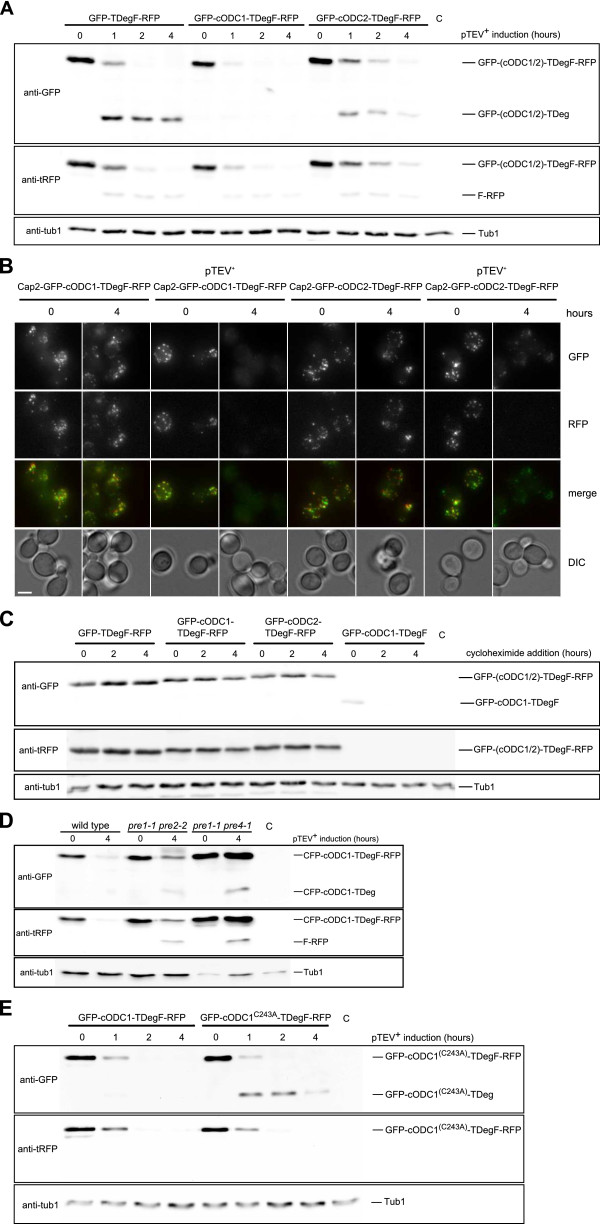
**Control of protein abundance using the bidirectional degron**. **(A) **The conditional C-degron included in cODC1-TDegF induces rapid protein depletion. The plasmid encoded constructs *GFP-TDegF-RFP*, *GFP-cODC1-TDegF-RFP*, and *GFP-cODC2-TDegF-RFP *were expressed in yeast cells (YCT1169) using the constitutive *ADH1 *promoter. Cells without a construct (lane C) served as control of antibody specificity. Expression of the *pTEV^+ ^protease *was induced by addition of galactose (2% final concentration). Samples of logarithmically growing yeast cells were taken at the indicated time points and subjected to western blotting. For detection, anti-GFP, anti-tRFP, and anti-tub1 (loading control) antibodies were used. Positions of cleaved and uncleaved species are indicated in the figure. **(B) **Observation of Cap2 depletion by live-cell imaging. *CAP2-GFP-cODC1-TDegF-RFP *or *CAP2-GFP-cODC2-TDegF-RFP *(chromosomally encoded) was expressed in strains containing or lacking the gene encoding for the pTEV^+ ^protease. Images (maximum intensity projections shown) were recorded before and after 4 hours of pTEV^+ ^protease production. Bar size, 2.5 μm. **(C) **Translation shut-off experiment to measure the destabilizing properties of the dormant degrons in GFP-TDegF-RFP, GFP-cODC1-TDegF-RFP, GFP-cODC2-TDegF-RFP, and GFP-cODC1-TDegF (plasmid encoded). The same conditions were used as described in A. Galactose and the translation elongation inhibitor cycloheximide were added at time point 0 hours. **(D) **Proteasomal activity is necessary for protein depletion mediated by the bidirectional degron. Plasmid encoded *CFP-cODC1-TDegF-RFP *was expressed in wild type and proteasomal mutant cells. Expression of the *pTEV^+ ^protease *(plasmid encoded) was induced by the addition of galactose. Cells were kept at 30°C during the experiment (semi-permissive conditions for the proteasomal mutants). Samples were prepared as described in A. **(E) **Destabilizing activity of the conditional C-degron in cODC1-TDegF depends on the cysteine-alanine motif. The plasmid encoded constructs GFP-cODC1-TDegF-RFP and GFP-cODC1^C243A^-TDegF-RFP were expressed in yeast cells using the constitutive *ADH1 *promoter. Experimental procedure as described in A.

We fused two different bidirectional degrons (cODC1/2-TDegF) between the green fluorescent protein (GFP) and the red fluorescent protein mKate (RFP) to obtain tester proteins. The behavior of the tester proteins was assessed upon *pTEV^+ ^protease *[25] expression. We observed complete cleavage of the tester proteins, depletion of the F-RFP as well as cODC-dependent destabilization of the GFP. Interestingly, we observed complete disappearance of the GFP signal only in case of the cODC1 construct (Figure 2A), showing that cODC1 mediates stronger destabilizing activity than cODC2.

For further characterization of the newly created C-degrons, we fused GFP-cODC1-TDegF-RFP and GFP-cODC2-TDegF-RFP to the C-terminus of Cap2. Both constructs localized to patches near the plasma membrane, mostly within the bud. This localization pattern was found for Cap2-GFP as well [35], showing that addition of the tags did not influence subcellular localization. We followed the GFP- and RFP- fluorescence by live-cell imaging in presence and absence of the pTEV^+ ^protease. Both, GFP- and RFP-fluorescence decreased in presence of the protease, almost complete loss of green fluorescence was observed in cells containing Cap2-GFP-cODC1-TDegF-RFP (Figure 2B). This demonstrates that membrane association of target proteins does not hamper proteolytic cleavage and depletion. A similar behavior has been found for substrates modified with the TDegF tag as well [25]. The cODC1-TDegF tag was constructed as a conditional degron. To clarify, whether the cODC sequences exert destabilizing activity on a protein without pTEV^+ ^protease cleavage, we assessed the stability of the tester proteins GFP-TDegF-RFP, GFP-cODC1-TDegF-RFP, GFP-cODC2-TDegF-RFP and GFP-cODC1-TDegF (a shortened construct lacking the RFP) after inhibition of protein synthesis. To do so, the cells were treated with the translation elongation inhibitor cycloheximide at the same time as production of the pTEV^+ ^protease was induced. As expected, the tester proteins were not processed due to absence of the protease. We observed no degradation of the tester proteins GFP-TDegF-RFP, GFP-cODC1-TDegF-RFP, and GFP-cODC2-TDegF-RFP, showing that the cODC sequences we used do not destabilize these constructs without activation of the degradation tag. However, no signal of the GFP-cODC1-TDegF construct was detected after cycloheximide treatment. Moreover, the steady-state level of this protein was reduced compared to the other constructs (Figure 2C). This indicates that the construct is destabilized without TEV protease cleavage. Presumably, this destabilization is induced by the largely unstructured SF3b155^381-424^-domain [36]. It is known that an unstructured domain serves as a weak signal for proteolysis by the ubiquitin proteasome system [37,38]. Although the SF3b155^381-424^-domain is present in all our tester proteins, destabilization occurs only in case of the GFP-cODC1-TDegF construct, which lacks a folded domain at the C-terminus of the protein.

Mouse ODC is degraded by the proteasome independently of the ubiquitin system [27]. Therefore, reduced proteasomal activity should lead to incomplete proteolysis of a cODC1-containing tester construct. Using the cyan fluorescent protein (CFP) containing tester construct CFP-cODC1-TDegF-RFP, we observed that *pTEV^+^ protease *expression led to accumulation of cleavage intermediates in *pre1-1 pre2-2 *or *pre1-1 pre4-1 *mutant cells [39,40] indicating incomplete proteolysis of CFP-cODC1-TDeg and F-RFP fragments (Figure 2D). Proteasomal degradation of mouse ODC depends on the cysteine-alanine motif, which is necessary for proteasomal association of the degron [26,29]. Mutation of this motif should abolish rapid depletion of cleaved GFP-cODC1-TDegF-RFP. We replaced the cysteine by an alanine and compared the behavior of this construct with GFP-cODC1-TDegF-RFP. As expected, we found complete depletion of GFP-cODC1-TDeg after pTEV^+ ^protease cleavage, whereas GFP-cODC1^C243A^-TDeg was much less prone to degradation (Figure 2E). The dependency on proteasomal activity and on the cysteine-alanine motif demonstrates that the cODC1 degron induces proteolysis by the proteasome. In addition, we performed flow cytometry measurements of cells expressing tester constructs (see Additional file 1 Figure S1A, B). These experiments confirmed our results obtained by immunoblotting (Figure 2A, C, E).

**Figure 3:**
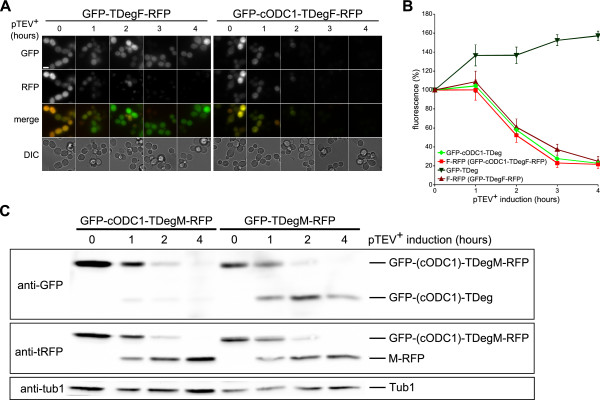
**Control of protein stability using combinations of stabilizing and destabilizing sequences**. **(A) **Live-cell imaging of cODC1-mediated destabilization of GFP. Plasmid encoded *GFP-TDegF-RFP *and *GFP-cODC1-TDegF-RFP *were expressed constitutively under control of the *ADH1 *promoter in yeast cells (YCT1169). Images of the cells were taken at the indicated time points after induction of *pTEV^+^ protease *expression. Bar size, 5 μm. **(B) **Kinetics of cODC1-mediated destabilization of GFP. Fluorescence intensities of the fragments, which were generated by TEV protease cleavage, were plotted over time. Images recorded for the experiment shown in A were used for automated quantitative image analysis to measure intracellular GFP and RFP fluorescence in 1000 to 3000 cells per strain (error bars indicate the standard error of the mean). The fragment GFP-TDeg is marked by inverted triangles, F-RFP (derived from GFP-TDegF-RFP) by triangles, GFP-cODC1-TDeg by diamonds, and F-RFP (derived from GFP-cODC1-TDegF-RFP) by squares. **(C) **Processing of tester proteins by the pTEV^+ ^protease. The plasmid encoded constructs *GFP-cODC1-TDegM-RFP *and *GFP-TDegM-RFP *were expressed in yeast cells (YCT1169) using the constitutive *ADH1 *promoter. Samples of logarithmically growing yeast cells were taken at the indicated time points after induction of *pTEV^+^ protease *expression and subjected to western blotting. For detection, anti-GFP, anti-tRFP, and anti-tub1 (loading control) antibodies were used. Positions of cleaved and uncleaved species are indicated in the figure.

Next, we compared the destabilizing activity of the active cODC1 degron to that of the N-degron TDegF. We performed fluorescence microscopy to visualize the GFP and RFP fluorescence of the constructs GFP-cODC1-TDegF-RFP and GFP-TDegF-RFP after pTEV^+ ^protease cleavage. Only cells containing the cODC1 construct showed rapid loss of GFP fluorescence, whereas RFP fluorescence decreased in both cases (Figure 3A). Quantification of the images revealed that GFP-cODC1-TDeg was depleted as quickly as F-RFP (Figure 3B). Our experiments with diverse tester proteins demonstrate that the bidirectional degron cODC1-TDegF induces rapid target protein depletion after TEV protease cleavage regardless if placed at the N- or C-terminus or inside the protein.

As shown in Figure 1D, the TIPI system is not restricted to complete destabilization of proteins. Constructs containing cODC1-TDegM and TDegM could be used to degrade only a single domain and to separate two domains without destabilization, respectively. As expected, proteolytic cleavage of GFP-cODC1-TDegM-RFP resulted in destabilized GFP-cODC1-TDeg and stable M-RFP whereas GFP-TDegM-RFP was split in two stable fluorescent proteins (Figure 3C).

In summary, our results demonstrate that it is possible to create quasi artificial, conditional degrons with distinct properties based on the features of the cODC degron. The newly developed cODC degrons induce rapid degradation and their destabilizing activities depend solely on proteolytic cleavage of the bidirectional degron by the pTEV^+ ^protease. The less destabilizing cODC2-TDegF tag might be useful to reduce target protein levels without complete depletion. Weak degrons have been used before to measure transcription [41] or proteasomal activity [42]. The data obtained with combinations of stabilizing sequences, N- or C-degrons (Figure 2A and 3C) show that the TIPI system provides a toolbox to control the fate of single domains *in vivo*.

### Creation of conditional mutants

**Figure 4:**
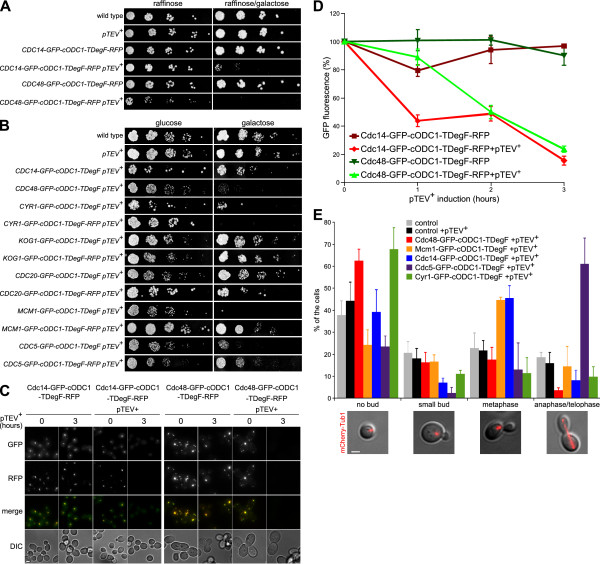
**Depletion of essential yeast proteins using the GFP-cODC1-TDegF-RFP and GFP-cODC1-TDegF tag causes phenotypes close to deletion mutants**. **(A) **The *GFP-cODC1-TDegF-RFP *tag was fused to the 3'-end of *CDC14 *and *CDC48 *in strains with or without the gene encoding for the pTEV+ protease. Serial dilutions (1:5) of cells were spotted on agar plates supplemented with either raffinose or galactose and raffinose and incubated at 30°C for 3 days. **(B) **The *GFP-cODC1-TDegF *tag or the *GFP-cODC1-TDegF-RFP *tag were fused to the 3'-end of *CDC14*, *CDC48, CYR1, KOG1*, *CDC20, MCM1*, and *CDC5 *in strains with the gene encoding for the pTEV^+ ^protease. Serial dilutions (1:5) of cells were spotted on agar plates supplemented with either glucose or galactose and incubated at 30°C for 3 days. **(C) **Cdc14-GFP-cODC1-TDegF-RFP and Cdc48-GFP-cODC1-TDegF-RFP are depleted quickly after *pTEV^+ ^protease *expression. The *GFP-cODC1-TDegF-RFP *tag was inserted at the 3'-end of *CDC14 *and *CDC48 *in yeast strains ESM356-1 and YCT1169. Maximum intensity projection images are shown. The Images were recorded at the indicated time points after induction of pTEV^+ ^protease production. Bar size, 2 μm. **(D) **Kinetics of Cdc14-GFP-cODC1-TDegF-RFP and Cdc48-GFP-cODC1-TDegF-RFP depletion. Automated quantitative image analysis was used to measure the cellular fluorescence of the green fluorescent protein in 1000 to 3000 cells per strain (error bars represent the standard error of the mean). Images recorded for the experiment shown in C were used for quantification. **(E) **Depletion of Cdc48-, Mcm1-, Cdc14-, Cdc5-, and Cyr1-GFP-cODC1-TDegF leads to cell-cycle defects. Cell-cycle stages were assessed after 4 hours of *pTEV^+ ^protease *expression. The bud size and spindle morphology were taken into account to classify the cells. Wild-type cells with and without expression of *pTEV^+ ^protease *were used as controls. Bar size, 2 μm.

We assessed, whether activation of the bidirectional degron induces depletion of target proteins to levels, at which specific phenotypes become observable. Therefore, we inserted the *GFP-cODC1-TDegF-RFP *tag and the *GFP-cODC1-TDegF* tag chromosomally at the 3' end of the essential genes *CDC14*, *CDC48, CYR1, KOG1, CDC20, MCM1*, and *CDC5*. The amino acid sequence of both tags is given in Figure S3 (see Additional file 1). We obtained conditional mutants for 5 of the 7 genes. No reduction of viability at restrictive conditions was observed for modified Kog1 and Cdc20, whereas GFP-cODC1-TDegF-RFP and GFP-cODC1-TDegF-tagged Cdc48 and Cyr1 as well as Mcm1-GFP-cODC1-TDegF, Cdc5-GFP-cODC1-TDegF, and Cdc14-GFP-cODC1-TDegF-RFP led to severe growth defects (Figure 4A, B). This demonstrates that the GFP-cODC1-TDegF-RFP and GFP-cODC1-TDegF tags can be used to create conditional mutants. A possible explanation for the differences between GFP-cODC1-TDegF-RFP and GFP-cODC1-TDegF mutants might lie in changes of target protein levels induced by the GFP-cODC1-TDegF tag at permissive conditions, as we found that the GFP-cODC1-TDegF construct is destabilized without TEV protease cleavage (Figure 2C). Indeed, steady state levels of the essential proteins modified with the GFP-cODC1-TDegF tag were reduced in comparison with the corresponding GFP-tagged proteins (data not shown). However, growth of the mutants was only slightly affected at permissive conditions on glucose containing media (Figure 4B). A reason could be that most yeast proteins are synthesized at much higher levels than necessary for survival under lab conditions [43].

Additionally, we followed the cleavage of the fusion proteins by the pTEV^+ ^protease using immunoblotting. We observed cleavage in case of Cdc5, Cdc14, Cdc48, Cyr1, and Mcm1 (see Additional file 1 Figure S2). Due to the absence of clear signals for Cdc20-, or Kog1-GFP-cODC1-TDegF (data not shown), it is not clear why creation of conditional mutants failed for these proteins.

We performed live-cell imaging with cells expressing Cdc14-GFP-cODC1-TDegF-RFP and Cdc48-GFP-cODC1-TDegF-RFP fusion proteins. The Cdc14-GFP-cODC1-TDegF-RFP fusion protein localized to the nucleolus, as expected, whereas Cdc48-GFP-cODC1-TDegF-RFP mislocalized to several very bright spots (Figure 4C). The GFP and RFP fluorescence dropped down to roughly 10% of the initial levels in both cases within three hours after *pTEV^+ ^protease *expression. The fluorescence did not change considerably in the absence of the protease (Figure 4C, D).

We checked the consequences of Cdc5, Cdc14, Cdc48, Cyr1, and Mcm1 depletion *in vivo*. All these proteins have essential functions during the yeast cell cycle, which were studied intensively with conventional mutants. Loss of activity results in each case in a specific defect. It was found that *cyr1-1 *and *cdc48-td *mutants arrest with no bud at restrictive conditions [44,45]. A metaphase arrest was observed in the *mcm1-110L *mutant [46] as well as the *TDegF-cdc14 *mutant [25] and an anaphase arrest in case of *cdc5 *temperature-sensitive mutants [47]. To compare the phenotypes obtained by protein depletion with conventional mutants, we checked for cell cycle phenotypes based on spindle morphology and bud-size in *GFP-cODC1-TDegF *mutants of *CDC5*, *CDC14*, *CDC48*, *CYR1 *and *MCM1*. We found that all *GFP-cODC1-TDegF *mutants accumulated in a specific cell cycle stage if the pTEV^+ ^protease was present (Figure 4E), matching the results, which were reported for other conditional mutants in the literature. In the absence of the pTEV^+ ^protease, we found no apparent difference to control cells (data not shown). Our data demonstrates that both, the GFP-cODC1-TDegF-RFP and the GFP-cODC1-TDegF tag can be used to create conditional mutants. In addition, the GFP-cODC1-TDegF-RFP tag is useful for applications, which ask for observation of substrate cleavage using live-cell imaging.

### Creation of developmental process-specific mutants

**Figure 5:**
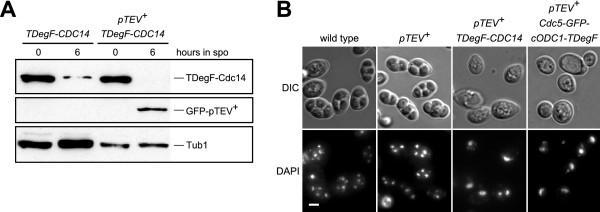
**Developmental process-specific depletion of essential proteins**. **(A) **Meiosis-specific depletion of Cdc14. Yeast cells with *P_CYC1_-GFP-TDegF-CDC14 *were subjected to sporulation in the absence or presence of *P_IME2_-GFP-pTEV^+^*. Samples were taken at the indicated time points after induction of meiosis due to shift on sporulation medium (spo). The proteins (GFP-TDegF-Cdc14 and GFP-pTEV^+ ^protease) were detected with antibodies directed against GFP; tubulin was used as loading control. **(B) **Depletion of Cdc14 or Cdc5 leads to a block during meiosis I. Yeast cells sporulated for 24 hours were stained with Hoechst 33342 to visualize the DNA. Bright field (DIC) and fluorescent (DAPI) images (maximum intensity projections) are shown. The genotypes of the strains are indicated in the figure. The *CDC5-GFP-cODC1-TDegF *cells contained a high copy plasmid with *P_IME2_-GFP-pTEV^+^*. Bar size, 2 μm.

Another interesting application for the TIPI system would be to use it as a method to deplete target proteins at a specific stage during a developmental process. We chose the developmental program of sporulation in *S. cerevisiae *as model process. Sporulation is initiated by starvation in yeast and consists of the meiotic cell divisions followed by spore formation [48]. To restrict the expression of the *pTEV^+ ^protease *to meiosis, we used the *IME2 *promoter, which is active only during sporulation [49]. As it is known that loss of Cdc14 or Cdc5 results in defects during the first meiotic division [50,51], we selected them as target proteins for N-degron and C-degron dependent depletion, respectively. We fused the *TDegF *tag under control of the *CYC1 *promoter, which is repressed during meiosis and spore formation [52], to the 5'-end of *CDC14*. This led to reduction of GFP-TDegF-Cdc14 levels, whereas additional expression of the *pTEV^+ ^protease *led to complete absence (Figure 5A) and a defect in spore formation, most likely due to a block in the first meiotic division (Figure 5B). We added the *GFP-cODC1-TDegF *tag to the 3'-end of *CDC5 *and subjected the cells to sporulation conditions. Surprisingly, the Cdc5-GFP-cODC1-TDegF containing cells sporulated in the presence of the pTEV^+ ^protease (data not shown). However, high-level production of the pTEV^+ ^protease achieved by transformation of the cells with a high-copy plasmid containing the *pTEV^+ ^protease *gene resulted in a block of sporulation in the meiotic prophase (Figure 5B). High-level production of the pTEV^+ ^protease alone did not change sporulation behavior of wild type yeast cells (data not shown). Thus, the TIPI system is a valuable technique to study developmental processes. Timely expression of the *TEV protease *during a specific cell cycle stage or a developmental process allows control of target protein abundance during that stage.

## Conclusions

The bidirectional degron can be fused to a target protein N-terminally, C-terminally or placed internally. Activation of the degrons is achieved by cleavage with the tobacco etch virus protease, resulting in quick degradation of the target protein. Thus the method provides a high degree of freedom to the user in terms of target protein modification with the bidirectional degron and control of TEV protease production. However, the usage might be constrained for some applications. The GFP-cODC1-TDegF-RFP tag is rather big as it contains two different fluorescent proteins. On the one hand this allows following cleavage of the tag by live-cell imaging, but on the other hand it limits the repertoire of additional fluorophores. The smaller GFP-cODC1-TDegF tag, which lacks the RFP, destabilizes the target protein in the absence of the TEV protease resulting in a reduction of protein levels. Even some of the GFP-cODC1-TDegF-RFP-tagged proteins exhibited reduced steady state levels (data not shown), but less pronounced than in case of the GFP-cODC1-TDegF-modified proteins. As we did not observe destabilization of the GFP-cODC1-TDegF-RFP tester protein without TEV protease cleavage (Figure 2C), this decrease might be caused by the leakiness of the *GAL1 *promoter [53] resulting in weak expression of the *TEV protease*. Another possible explanation might be that the GFP-cODC1-TDegF-RFP tag changes the efficiency of protein synthesis. The normal or only slightly affected growth of the conditional mutants at permissive conditions (Figure 4A, B) could be explained by the observation that most yeast proteins are synthesized in excess [43]. A new construct, lacking the GFP and keeping the RFP, might be a way to decrease the size of the construct and to circumvent the problem of reduced protein abundance.

The C- and N-degron we used to construct the bidirectional degron are based on conserved degradation mechanisms and are known to work in higher eukaryotes [17,54]. Therefore, protein depletion utilizing the TIPI system should be possible in animals as well, as it has been shown already that cell type specific usage of the TEV protease in *Drosophila melanogaster *is feasible [55]. In synthetic biology approaches the TIPI system might be employed to implement protein destabilization into regulatory circuits or to cleave a protein artificially. Advantageous is that the system is reversible [25] and allows genetic control of protein stability. Furthermore, the degradation tag and the TEV protease are heterologous proteins or protein domains. Therefore, potentially harmful interactions with cellular components are minimized. Previously, chromosome separation has been studied using TEV protease cleavage [55,56], but without incorporation of conditional degrons. Our work enhances the existing techniques, as it provides the possibility to inflict different fates to the N- and C-terminus of the target protein after cleavage. Destabilization of one or the other is achieved using the right combination of degron and stabilizing sequence (Figure 1D). This might be a way to probe for regulatory domains or to create separation-of-function-mutants at protein level. These applications are unique to the TIPI system and exemplify its value as a cell biology tool to manipulate protein abundance and activity *in vivo*.

## Methods

### Yeast strains, growth conditions and plasmid constructions

The yeast strains were derived from the S288C strain ESM356-1 [57], WCG4a [40] or the SK-1 strain YKS32 [58] as indicated in the Table S1 (see Additional file 2). Standard methods were used to construct yeast strains; standard preparations of media were used for growth [59]. Gene tagging with PCR products was performed as described [53]. Yeast cells were grown at 30°C, cells used for the experiment shown in Figure 2D (WCG4a, WCG4a-11/22, and YHI29/14) were grown at 25°C and shifted to 30°C for the time of the experiment. Growth tests were performed on rich media supplemented with glucose, raffinose or galactose (2% final concentration), as indicated for the experiments. Cells were grown in low-fluorescence media [60] for fluorescent microscopy experiments. Plasmids were constructed by standard procedures [61], details and sequences of the used vectors are available on request. The plasmids are listed in Table S2 (see Additional file 2).

### Immunoblotting and cycloheximide chase experiment

The immunoblotting experiments were performed essentially as described [25]. The amount of yeast culture corresponding to 1 OD_600 _of cells was collected at each time point. Cells were grown in synthetic complete media supplemented with raffinose (2%), galactose was added after collection of the first sample (time 0 hours) to induce the pTEV^+ ^protease production. Samples were subjected to alkaline lysis and TCA precipitation, SDS-PAGE and blotting. Commercially available antibodies against GFP (Santa Cruz biotechnology, Santa Cruz, USA), tRFP (Biocat, Heidelberg, Germany) and HRPO-coupled antibodies directed against mouse or rabbit (Santa Cruz biotechnology, Santa Cruz, USA) were used to detect tester proteins. The rabbit anti-tubulin antibody (a kind gift of M. Knop, EMBL Heidelberg) was used to detect tubulin, which served as a loading control. Chemiluminescence was detected using a western blot imager (INTAS Science Imaging Instruments, Göttingen, Germany). Signal intensities were quantified with the software ImageJ [62]. To perform the translation inhibition experiment (Figure 2C), cycloheximide was added to the cultures at a final concentration of 0.1 mg/ml. Samples were collected at the times indicated in the figure and subjected to alkaline lysis and TCA precipitation.

### Microscopy

Live-cell imaging was performed as described [25,60] using a Zeiss Axiovert 200 equipped with a Hamamatsu camera, DAPI, EGFP and rhodamine filter sets and a 63 times Plan Apochromat oil lens (NA 1.4). Images were collected as z-stacks with 0.5 μm spacing (Figure 2B, 4C) or single images (Figure 3A). Quantitative imaging was performed as follows: Logarithmically grown yeast cells were adhered to glass-bottom-dishes (MatTek Corporation, Ashland, USA) treated with concanavalin A. Synthetic complete media with raffinose was added and z-stack images with 0.5 μm spacing or single images were taken at the time points indicated in the figures. Galactose (2% final concentration) was added to the medium after collection of the 0 hours image series. Images of the cells were collected in the bright field and fluorescent channels indicated in the figures. Quantification of the images was done with the software ImageJ (version 1.40g). Slightly out-of-focus bright field images were used to detect the cell outlines. The commands "find edges", "gaussian blur (radius = 2)", "contrast setting" and "make binary" were used on these images to generate binary images of cell outlines. With the commands "fill holes" and "create selection" a mask covering whole cells was obtained. These masks were used to measure mean signal intensities in the images of the fluorescence channels. Maximum projections of z-stacks were used for the measurements in case z-stack images were recorded.

### Growth assays

Serial dilutions (1:5) of cells grown in YP + raffinose or YPD were placed on YP + raffinose, YP + raffinose and galactose, YPD or YP + galactose plates (as indicated in Figure 4). Cells corresponding to 0.05 OD_600 _were diluted in 1 ml of water. Subsequently, 4 dilutions were made and 3 μl of each dilution were spotted on the plates. Pictures of the spots were taken after incubation for 3 days at 30°C.

### FACS measurements

The cells were grown to mid-log phase in low fluorescence media containing raffinose. The culture was divided in two; one was treated with sodium azide (10 mM final concentration) and kept on ice until the measurement in the flow cytometer (sample -TEV). The other half of the culture was supplemented with galactose. The cells were incubated with shaking for 4 hours at 30°C and sodium azide was added (sample +TEV). The cells were analyzed in a FACSCalibur flow cytometer (Becton Dickinson) equipped with a 488 nm argon laser and a 530/30 band-pass filter for GFP fluorescence detection. Around 85 000 events were collected for each sample; all events were used to create the graphs and to calculate the median fluorescence.

### Sporulation

Synchronous sporulation was performed using the presporulation treatment as described [63]. Potassium acetate (1%) was used as sporulation medium. Cells were stained with Hoechst 33342 after fixation with 70% ethanol.

## Authors' contributions

All authors were involved in generation of plasmids and yeast strains. MJ and CR performed the sporulation experiments and helped to write the manuscript. CT conceived the study, carried out experiments and wrote the manuscript. All authors read and approved the final manuscript.

## Supplementary Material

Additional file 1 **FACS measurements of tester constructs, depletion of essential yeast proteins and sequence of the GFP-cODC1-TDegF and GFP-cODC1-TDegF-RFP tags**. *FACS measurements*. We performed flow cytometry measurements with cells expressing GFP-TDegF-RFP, GFP-cODC1-TDegF-RFP, GFP-cODC1^C243A^-TDegF-RFP, GFP-cODC2-TDegF-RFP, and GFP-cODC1-TDegF to measure the GFP fluorescence in the absence and the presence of the pTEV^+ ^protease. We found background fluorescence in the absence of the protease in case of the GFP-cODC1-TDegF construct. This argues for a destabilization of the construct independently of the cODC degron. Nevertheless, we detected robust GFP fluorescence in the other constructs in the absence of the protease. Cells producing the pTEV^+ ^protease showed nearly background fluorescence in the cODC containing constructs and reduced GFP fluorescence in case of the constructs GFP-TDegF-RFP and GFP-cODC1^C243A^-TDegF-RFP (Figure S1A, B). *Depletion of essential yeast proteins*. We checked the production and proteolytic cleavage of the fusion proteins Cdc14- and Cdc48-GFP-cODC1-TDegF-RFP as well as the Cdc5-, Cyr1-, and Mcm1-GFP-cODC1-TDegF by immunoblotting. It was possible to detect all fusion proteins in crude yeast extracts in the absence of the pTEV^+ ^protease. Presence of the protease resulted in partial or complete depletion of the target proteins (Figure S2). *Sequence of the GFP-cODC1-TDegF and GFP-cODC1-TDegF-RFP tags*. The amino acid sequence of the GFP-cODC1-TDegF and the GFP-cODC1-TDegF-RFP tags is given (Figure S3). The sequences of yeast enhanced GFP and mKate have been published [64,65]. Click here for file

Additional file 2 **Information on yeast strains (Table S1) and plasmids (Table S2) used for this study**. Click here for file
