# Supplementary material for: Targeted protein depletion in Saccharomyces cerevisiae by activation of a bidirectional degron
Source: BMC Syst Biol. 2010 Dec 29;4:176. doi: 10.1186/1752-0509-4-176 (PMC3024245; doi:10.1186/1752-0509-4-176)
Supplement: Additional file 1 — FACS measurements of tester constructs, depletion of essential yeast proteins and sequence of the GFP-cODC1-TDegF and GFP-cODC1-TDegF-RFP tags. FACS measurements. We performed flow cytometry measurements with cells expressing GFP-TDegF-RFP, GFP-cODC1-TDegF-RFP, GFP-cODC1C243A-TDegF-RFP, GFP-cODC2-TDegF-RFP, and GFP-cODC1-TDegF to measure the GFP fluorescence in the absence and the presence of the pTEV+ protease. We found background fluorescence in the absence of the protease in case of the GFP-cODC1-TDegF construct. This argues for a destabilization of the construct independently of the cODC degron. Nevertheless, we detected robust GFP fluorescence in the other constructs in the absence of the protease. Cells producing the pTEV+ protease showed nearly background fluorescence in the cODC containing constructs and reduced GFP fluorescence in case of the constructs GFP-TDegF-RFP and GFP-cODC1C243A-TDegF-RFP (Figure S1A, B). Depletion of essential yeast proteins. We checked the production and proteolytic cleavage of the fusion proteins Cdc14- and Cdc48-GFP-cODC1-TDegF-RFP as well as the Cdc5-, Cyr1-, and Mcm1-GFP-cODC1-TDegF by immunoblotting. It was possible to detect all fusion proteins in crude yeast extracts in the absence of the pTEV+ protease. Presence of the protease resulted in partial or complete depletion of the target proteins (Figure S2). Sequence of the GFP-cODC1-TDegF and GFP-cODC1-TDegF-RFP tags. The amino acid sequence of the GFP-cODC1-TDegF and the GFP-cODC1-TDegF-RFP tags is given (Figure S3). The sequences of yeast enhanced GFP and mKate have been published [64,65]. [file 1752-0509-4-176-S1.PDF]

Additional File 1
Figure S1

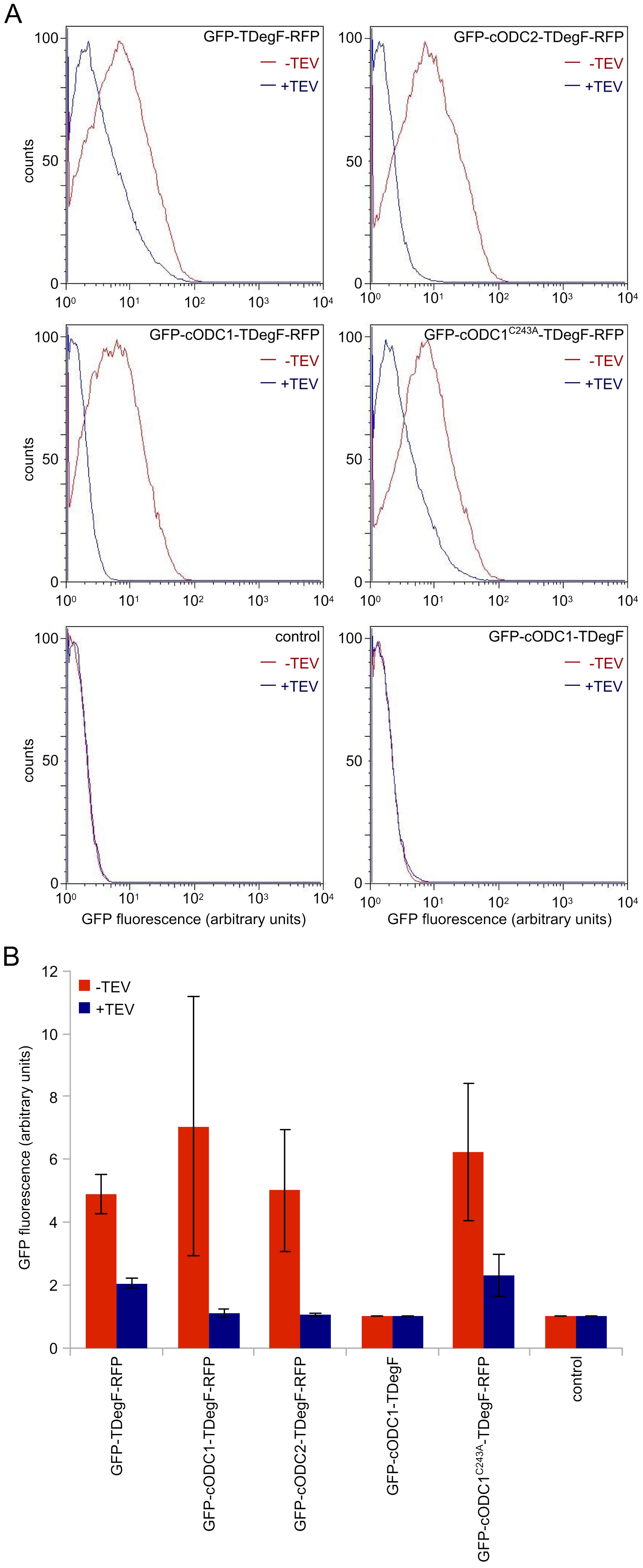

Figure S1

(A) Flow cytometry measurements of GFP fluorescence. The plasmid encoded constructs *GFP-TDegF-RFP*, *GFP-cODC1-TDegF-RFP*, *GFP-cODC2-TDegF-RFP, GFP-cODC1*^{C243A}*-TDegF-RFP,* and *GFP-cODC1-TDegF* were expressed in yeast cells (YCT1169) using the constitutive *ADH1* promoter. Cells with an empty plasmid (pRS314) were used to measure autofluorescence. Each construct was measured twice from independently grown cultures. Representative graphs are shown for each construct. Cells were measured before (-TEV) and after 4 hours of pTEV+ protease production (+TEV).

(B) Median GFP fluorescence of the constructs measured for the experiment shown in A. Mean values of two measurements are shown for each construct (error bar: standard deviation).

Figure S2

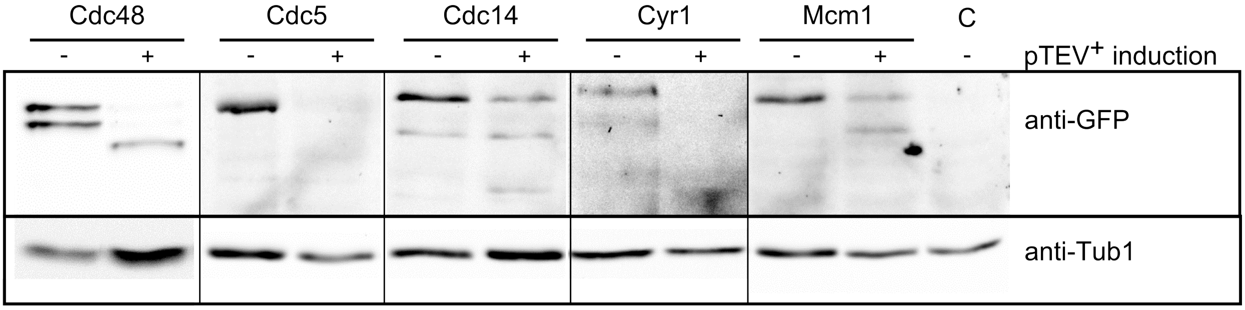

Figure S2

Destabilization of GFP-cODC1-TDegF-tagged proteins visualized by western blotting. Cells expressing the fusion proteins Cdc48-GFP-cODC1-TDegF-RFP, Cdc5-GFP-cODC1-TDegF, Cdc14-GFP-cODC1-TDegF-RFP, Cyr1-GFP-cODC1-TDegF and Mcm1- GFP-cODC1-TDegF were analyzed by western blotting before and 4 hours after induction of *pTEV*$^{+}$ *protease* expression. To generate the fusions, *GFP-cODC1-TDegF* or *GFP-cODC1-TDegF-RFP* was integrated chromosomally at the 3' end of *CDC48*, *CDC5*, *CDC14*, *CYR1*, and *MCM1*. Cells without a construct served as control of antibody specificity (C). For detection, anti-GFP, and anti-Tub1 (loading control) antibodies were used. Please note that the fusion proteins have different molecular weights. For reasons of simplicity, the bands were placed next to each other.

Figure S3

Sequence of the GFP-cODC1-TDegF-RFP and GFP-cODC1-TDegF tags. The end of the GFP-cODC1-TDegF tag is marked by a *. The cODC1 degron is highlighted in red, the spacer region in orange and the TEV protease cleavage site in green. The sequences of yeast enhanced GFP and mKate have been published [1, 2]

```
yeGFP...GITHGMDELYKLPMSCAQESITSLYKKAGSENLYFQFHKSGAWKLPVSLVKRGIDKLD
YKEQLQAWRWEREIDERNRPLSDEELDAMFPEGYKVLPPPAGYVPIRTPAHMDRIPAV*AGAGAGA
GAMSELIK...mKate
```

References

1. Cormack BP, Bertram G, Egerton M, Gow NA, Falkow S, Brown AJ: **Yeast-enhanced green fluorescent protein (yEGFP)a reporter of gene expression in Candida albicans.** *Microbiology* 1997, **143 (Pt 2):**303-311.

2. Shcherbo D, Merzlyak EM, Chepurnykh TV, Fradkov AF, Ermakova GV, Solovieva EA, Lukyanov KA, Bogdanova EA, Zaraisky AG, Lukyanov S, Chudakov DM: **Bright far-red fluorescent protein for whole-body imaging.** *Nat Methods* 2007, **4:**741-746.
