# Supplementary material for: Targeted protein depletion in Saccharomyces cerevisiae by activation of a bidirectional degron
Source: BMC Syst Biol. 2010 Dec 29;4:176. doi: 10.1186/1752-0509-4-176 (PMC3024245; doi:10.1186/1752-0509-4-176)
Supplement: Additional file 2 — Information on yeast strains (Table S1) and plasmids (Table S2) used for this study. [file 1752-0509-4-176-S2.PDF]

Additional File 2

Yeast strains used for the figures

Figure 2

(A) YCT1169 [pCT303]; YCT1169 [pDS38]; YCT1169 [pDS42]; YCT1169 (left to right)

(B) YCT1233; YCT1234; YCT1235; YCT1236 (left to right)

(C) YCT1169 [pCT303]; YCT1169 [pDS38]; YCT1169 [pDS42]; YCT1169 [pDS53]; YCT1169 (left to right)

(D) WCG4a [pDS5 pDS46]; WCG4/11/22 [pDS5 pDS46]; YHI29/14 [pDS5 pDS46]; WCG4a (left to right)

(E) YCT1169 [pDS38]; YCT1169 [pCT336]; YCT1169

Figure 3

(A) YCT1169 [pCT303]; YCT1169 [pDS38] (left to right)

(C) YCT1169 [pDS57]; YCT1169 [pDS58] (left to right)

Figure 4

(A) ESM356-1; YCT1169; YCT1247; YCT1248; YCT1249; YCT1250 (top to bottom)

(B) ESM356-1; YCT1244; YDS48; YCT1263; YCT1258; YCT1251; YCT1262; YCT1252; YCT1257; YCT1253; YCT1260; YCT1314; YCT1259; YCT1246 (top to bottom)

(C) YCT1248; YCT1247; YCT1250; YCT1249 (left to right)

(E) ESM356-1; YCT1169; YCT1264; YDS45; YDS48; YDS46; YDS47 (top to bottom)

Figure 5

(A) YCT1212; YCT1212 [pCR3] (left to right)

(B) YKS32; YMJ79; YDS12; YDS30 [pDS37] (left to right)

Figure S1

(A) YCT1169 [pCT303]; YCT1169 [pDS38]; YCT1169 [pRS314]; YCT1169 [pDS42]; YCT1169 [pCT336]; YCT1169 [pDS53] (top left to bottom right)

(B) YCT1169 [pCT303]; YCT1169 [pDS38]; YCT1169 [pDS42]; YCT1169 [pDS53]; YCT1169 [pCT336]; YCT1169 [pRS314] (left to right)

Figure S2

YCT1250; YCT1259; YCT1248; YCT1251; YCT1260; YCT1169 (left to right)

Table S1: yeast strains

Name	Genotype	Source
ESM356	*Mat a ura3-53 leu2Δ1 his3Δ200 trp1Δ63*	[1]
YCT1169	ESM356 *ura3::*P_{GAL1}*-p14*^{D122Y}*TEV*$^{S219V\ 234STOP}$*::kanMX*	[2]
YCT1244	ESM356 *ura3::*P_{GAL1}*-p14*^{D122Y}*-myc-TEV*$^{S219V\ 224STOP}$*::kanMX*	This work
YCT1233	ESM356 *CAP2-GFP-cODC1-TDegF-mKate::hphNT1*	This work
YCT1234	YCT1169 *CAP2-GFP-cODC1-TDegF-mKate::hphNT1*	This work
YCT1235	ESM356 *CAP2-GFP-cODC2-TDegF-mKate::hphNT1*	This work
YCT1236	YCT1169 *CAP2-GFP-cODC2-TDegF-mKate::hphNT1*	This work
YCT1246	YCT1169 *CDC5-GFP-cODC1-TDegF-mKate::hphNT1*	This work
YCT1247	ESM356 *CDC14-GFP-cODC1-TDegF-mKate::hphNT1*	This work
YCT1248	YCT1169 *CDC14-GFP-cODC1-TDegF-mKate::hphNT1*	This work
YCT1249	ESM356 *CDC48-GFP-cODC1-TDegF-mKate::hphNT1*	This work
YCT1250	YCT1169 *CDC14-GFP-cODC1-TDegF-mKate::hphNT1*	This work
YCT1251	YCT1169 *CYR1-GFP-cODC1-TDegF-mKate::hphNT1*	This work
YCT1252	YCT1169 *KOG1-GFP-cODC1-TDegF-mKate::hphNT1*	This work
YCT1253	YCT1169 *CDC20-GFP-cODC1-TDegF-mKate::hphNT1*	This work
YCT1257	YCT1244 *CDC20-GFP-cODC1-TDegF::hphNT1*	This work
YCT1258	YCT1244 *CYR1-GFP-cODC1-TDegF::hphNT1*	This work
YCT1259	YCT1244 *CDC5-GFP-cODC1-TDegF::hphNT1*	This work
YCT1260	YCT1244 *MCM1-GFP-cODC1-TDegF::hphNT1*	This work
YCT1262	YCT1244 *KOG1-GFP-cODC1-TDegF::hphNT1*	This work
YCT1263	YCT1244 *CDC48-GFP-cODC1-TDegF::hphNT1*	This work
YCT1314	YCT1169 *MCM1-GFP-cODC1-TDegF-mKate::hphNT1*	This work
YDS28	ESM356 P_{HIS3}*-mCherry-TUB1::TRP1*	This work
YDS29	YCT1169 P_{HIS3}*-mCherry-TUB1::TRP1*	This work
YCT1264	YCT1244 *CDC48-GFP-cODC1-TDegF::hphNT1* P_{HIS3}*-mCherry-TUB1::TRP1*	This work
YDS48	YCT1169 *CDC14-GFP-cODC1-TDegF::caURA3* P_{HIS3}*-mCherry-TUB1::TRP1*	This work
YDS45	YCT1169 *MCM1-GFP-cODC1-TDegF::hphNT1* P_{HIS3}*-mCherry-TUB1::TRP1*	This work
YDS46	YCT1169 *CDC5-GFP-cODC1-TDegF::hphNT1* P_{HIS3}*-mCherry-TUB1::TRP1*	This work
YDS47	YCT1169 *CYR1-GFP-cODC1-TDegF::hphNT1* P_{HIS3}*-mCherry-TUB1::TRP1*	This work
WCG4a	*Mata ura3 leu2-3,112 his3-11,15 CanS* Gal^{+}	[3]
WCG4/11/22	WCG4a *pre1-1 pre2-2*	[3]

Name	Genotype	Source
YHI29/14	WCG4a *pre1-1 pre4-1*	[4]
YKS32	Mat a/Mat α *lys2/lys2 ura3/ura3 leu2/LEU2 ho::hisG/ho::LYS*	[5]
YMJ79	YKS32 *ura3::*P_{IME2}*-p14*^{D122Y}*-TEV*$^{S219V\ 234STOP}$*::kanMX/ura3::*P_{IME2}*-p14*^{D122Y}*-TEV*$^{S219V\ 234STOP}$*::kanMX*	This work
YCT1212	YKS32 *natNT2::*P_{CYC1}*-GFP-TDegF-CDC14/natNT2::*P_{CYC1}*-GFP-TDegF-CDC14*	This work
YDS12	YMJ79 *natNT2::*P_{CYC1}*-GFP-TDegF-CDC14/natNT2::*P_{CYC1}*-GFP-TDegF-CDC14*	This work
YDS30	YMJ79 *CDC5-GFP-cODC1-TDegF::hphNT1/CDC5-GFP-cODC1-TDegF::hphNT1*	This work

Table S2: plasmids

Name	Features	Source
pCT271	*ura3::P*$_{GAL1}$*-p14*^{D122Y}*-TEV*$^{S219V\ 234STOP}$*::kanMX* in pRS306K	[2]
pDS15	*ura3::P*$_{GAL1}$*-p14*^{D122Y}*-myc-TEV*$^{S219V\ 224STOP}$*::kanMX* in pRS306K	This work
pDS5	*P*$_{GAL1}$*-yeCitrine-p14*^{D122Y}*-TEV*$^{S219V\ 234STOP}$ in pRS416	[2]
pCT314	*GFP-cODC1-TDegF-SF3B155*$^{381\text{-}424}$*-mKate::hphNT1*	This work
pCT315	*GFP-cODC2-TDegF-SF3B155*$^{381\text{-}424}$*-mKate::hphNT1*	This work
pDS41	*GFP-cODC1-TDegF-SF3B155*$^{381\text{-}424}$*::hphNT1*	This work
pCT321	*GFP-cODC1-TDegF-SF3B155*$^{381\text{-}424}$*::caURA3*	This work
pCT303	*P*$_{ADH1}$*-GFP-TDegF-SF3B155*$^{381\text{-}424}$*-mKate* in pRS314	This work
pDS38	*P*$_{ADH1}$*-GFP-cODC1-TDegF-SF3B155*$^{381\text{-}424}$*-mKate* in pRS314	This work
pDS42	*P*$_{ADH1}$*-GFP-cODC2-TDegF-SF3B155*$^{381\text{-}424}$*-mKate* in pRS314	This work
pDS53	*P*$_{ADH1}$*-GFP-cODC1-TDegF-SF3B155*$^{381\text{-}424}$ in pRS314	This work
pCT336	*P*$_{ADH1}$*-GFP-cODC1*^{C243A}*-TDegF-SF3B-mKATE* in pRS314	This work
pDS57	*P*$_{ADH1}$*-GFP-cODC1-TDegM-SF3B155*$^{381\text{-}424}$*-mKate* in pRS314	This work
pDS58	*P*$_{ADH1}$*-GFP-TDegM-SF3B155*$^{381\text{-}424}$*-mKate* in pRS314	This work
pDS46	*P*$_{ADH1}$*-CFP-cODC1-TDegF-SF3B155*$^{381\text{-}424}$*-mKate* in pRS313	This work
pAK10	*P*$_{HIS3}$*-mCherry-TUB1::TRP1* in pRS304	[6]
pMJ11	*ura3::P*$_{IME2}$*-p14*^{D122Y}*-TEVS*$^{219V\ 234STOP}$*::kanMX* in pRS306K	This work
pCR3	*P*$_{IME2}$*-GFP-p14*^{D122Y}*-TEV*$^{S219V\ 234STOP}$ in pRS41H	This work
pDS37	*P*$_{IME2}$*-GFP-p14*^{D122Y}*-TEV*$^{S219V\ 234STOP}$ in pRS426	This work
pRS416	*URA3 ARS/CEN*	[7]
pRS314	*TRP1 ARS/CEN*	[7]
pRS315	*LEU2 ARS/CEN*	[7]
pRS426	*URA3 2µ*	[8]
pRS41H	*hphNT1 ARS/CEN*	[9]

References

1. Pereira G, Tanaka TU, Nasmyth K, Schiebel E: **Modes of spindle pole body inheritance and segregation of the Bfa1p-Bub2p checkpoint protein complex.** *EMBO J* 2001, **20:**6359-6370.
2. Taxis C, Stier G, Spadaccini R, Knop M: **Efficient protein depletion by genetically controlled deprotection of a dormant N-degron.** *Mol Syst Biol* 2009, **5:**267.
3. Heinemeyer W, Gruhler A, Mohrle V, Mahe Y, Wolf DH: **PRE2, highly homologous to the**

human major histocompatibility complex-linked RING10 gene, codes for a yeast proteasome subunit necessary for chrymotryptic activity and degradation of ubiquitinated proteins. *J Biol Chem* 1993, **268:**5115-5120.
4. Gerlinger UM, Guckel R, Hoffmann M, Wolf DH, Hilt W: **Yeast cycloheximide-resistant crl mutants are proteasome mutants defective in protein degradation.** *Mol Biol Cell* 1997, **8:**2487-2499.
5. Knop M, Strasser K: **Role of the spindle pole body of yeast in mediating assembly of the prospore membrane during meiosis.** *EMBO J* 2000, **19:**3657-3667.
6. Khmelinskii A, Lawrence C, Roostalu J, Schiebel E: **Cdc14-regulated midzone assembly controls anaphase B.** *J Cell Biol* 2007, **177:**981-993.
7. Sikorski RS, Hieter P: **A system of shuttle vectors and yeast host strains designed for efficient manipulation of DNA in Saccharomyces cerevisiae.** *Genetics* 1989, **122:**19-27.
8. Christianson TW, Sikorski RS, Dante M, Shero JH, Hieter P: **Multifunctional yeast high-copy-number shuttle vectors.** *Gene* 1992, **110:**119-122.
9. Taxis C, Knop M: **System of centromeric, episomal, and integrative vectors based on drug resistance markers for Saccharomyces cerevisiae.** *Biotechniques* 2006, **40:**73-78.
